# Aging-relevant human basal forebrain cholinergic neurons as a cell model for Alzheimer’s disease

**DOI:** 10.1186/s13024-020-00411-6

**Published:** 2020-10-21

**Authors:** Shuaipeng Ma, Tong Zang, Meng-Lu Liu, Chun-Li Zhang

**Affiliations:** 1 Department of Molecular Biology, University of Texas Southwestern Medical Center, 6000 Harry Hines Boulevard, Dallas, TX 75390 USA; 2 Hamon Center for Regenerative Science and Medicine, University of Texas Southwestern Medical Center, 6000 Harry Hines Boulevard, Dallas, TX 75390 USA

**Keywords:** Alzheimer’s disease, Aging, BFCNs, Reprogramming, Fibroblasts, Nucleocytoplasmic transport, TAU hyperphosphorylation

## Abstract

**Background:**

Alzheimer’s disease (AD) is an adult-onset mental disorder with aging as a major risk factor. Early and progressive degeneration of basal forebrain cholinergic neurons (BFCNs) contributes substantially to cognitive impairments of AD. An aging-relevant cell model of BFCNs will critically help understand AD and identify potential therapeutics. Recent studies demonstrate that induced neurons directly reprogrammed from adult human skin fibroblasts retain aging-associated features. However, human induced BFCNs (hiBFCNs) have yet to be achieved.

**Methods:**

We examined a reprogramming procedure for the generation of aging-relevant hiBFCNs through virus-mediated expression of fate-determining transcription factors. Skin fibroblasts were obtained from healthy young persons, healthy adults and sporadic AD patients. Properties of the induced neurons were examined by immunocytochemistry, qRT-PCR, western blotting, and electrophysiology.

**Results:**

We established a protocol for efficient generation of hiBFCNs from adult human skin fibroblasts. They show electrophysiological properties of mature neurons and express BFCN-specific markers, such as CHAT, p75NTR, ISL1, and VACHT. As a proof-of-concept, our preliminary results further reveal that hiBFCNs from sporadic AD patients exhibit time-dependent TAU hyperphosphorylation in the soma and dysfunctional nucleocytoplasmic transport activities.

**Conclusions:**

Aging-relevant BFCNs can be directly reprogrammed from human skin fibroblasts of healthy adults and sporadic AD patients. They show promises as an aging-relevant cell model for understanding AD pathology and may be employed for therapeutics identification for AD.

## Background

The basal forebrain cholinergic system, located to the front of and below the striatum, is the predominant source of cortical cholinergic input [1]. Early and progressive degeneration of basal forebrain cholinergic neurons (BFCNs) contributes substantially to cognitive impairments of human patients with Alzheimer’s disease (AD) [2, 3]. The importance of BFCNs in AD is further demonstrated in animal models, the behavior of which can be significantly improved through cell grafts [4, 5] or treatments promoting BFCN function [6]. As such, cell models of human BFCNs will be invaluable in understanding AD and identifying novel therapeutics.

BFCNs can be derived from embryonic stem cells (ESCs) or induced pluripotent stem cells (iPSCs) through sequential treatments with signaling molecules [4, 5, 7–13]. However, neurons derived from ESCs or iPSCs are young and do not exhibit aging-associated features [9, 14–17]. These young neurons are advantageous in understanding neural development and for transplantation-based therapies [4, 5, 7, 11, 12]. However, they may not be appropriate as a cell model for late-onset neurodegeneration, such as AD, a majority of which are sporadic without any known genetic mutations. We and others have recently shown that neurons can be directly reprogrammed from skin fibroblasts of adult human patients [15–20]. These induced neurons do not pass through an age-resetting progenitor stage and therefore retain aging-associated features, such as DNA damages, chromatin structures and nuclear organization, and increased biomarker of cellular senescence [15–17]. To date, however, human induced BFCNs (hiBFCNs) from adult skin fibroblasts have not been reported.

Here, we report a protocol for direct reprogramming of adult human skin fibroblasts into electrophysiologically mature hiBFCNs. The reprogramming efficiency is similar between fibroblasts of healthy and sporadic AD patients. The reprogrammed neurons retain aging-associated features. Our preliminary results further indicate that hiBFCNs from sporadic AD patients exhibit time-dependent TAU hyperphosphorylation and impairment in nucleocytoplasmic transport. hiBFCNs may be useful for understanding the molecular mechanisms and discovering novel therapeutics for age-dependent progressive AD.

## Results

### Rapid and efficient generation of hiBFCNs from adult human skin fibroblasts

**Fig. 1:**
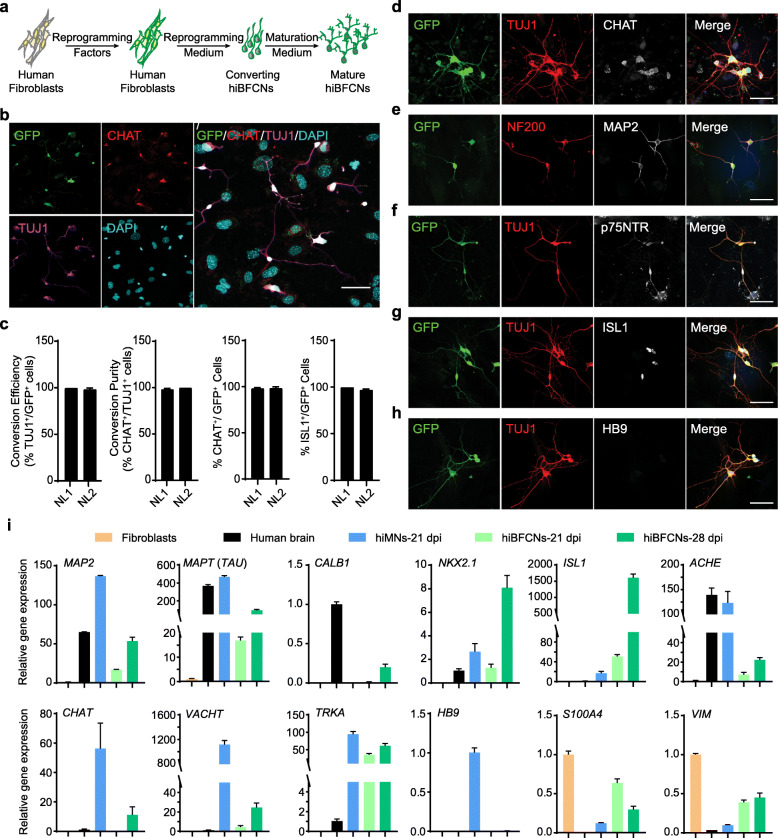
Direct induction of BFCNs from adult human skin fibroblasts. **a** A schematic representation of the reprogramming procedure. **b** Confocal images showing marker expression in hiBFCNs at 28 dpi. The virus-transduced cells are indicated by GFP fluorescence. Nuclei are counterstained with DAPI and include hiBFCNs and the co-cultured astrocytes. Scale bar, 50 μm. **c** Quantification of the reprogramming efficiency and neuronal purity. Cells were co-cultured with primary astrocytes and analyzed at 28 dpi (mean ± SEM; *n* = 3 independent samples; 10 randomly selected 20× fields per sample were examined). **d**-**h** Confocal images showing expression of the indicated markers in hiBFCNs co-cultured with astrocytes at 28 dpi. hiBFCNs do not express HB9 (**h**), a marker restricted to cholinergic motor neurons. Scale bar, 50 μm. **i** Marker expression by qRT-PCR analysis. Samples from fibroblasts, human brains, and hiMNs were used as controls. All gene expression was normalized to *GAPDH*

We previously showed that human skin fibroblasts can be directly converted into cholinergic neurons without passing through a progenitor stage [18]. However, they lack the expression of LHX8 (also known as LHX7 or L3 [21]) and GBX1, transcription factors crucial for BFCN specification [7, 12, 22–26]. We then examined these two transcription factors in various combinations with our original reprogramming factors (NEUROG2 and SOX11) for cholinergic neurons [18]. Two days post-viral infection (dpi), transduced cells were switched to neuron-induction medium [18, 19]. Neuronal conversion was monitored daily by live-cell fluorescence microscopy. Cells were replated at 14 dpi to remove most non-reprogrammed fibroblasts and were seeded into astrocytes-coated plates with maturation medium for long-term survival (Fig. 1a).

Although induced neurons (iNs) could be obtained from normal (NL) healthy patient fibroblasts, some of them expressed HB9, a transcription factor specifically expressed in spinal motor neurons. This was likely due to a dominant role of NEUROG2 in motor neuron specification [18, 27, 28], whereas SOX11 promotes neuronal survival but not fate reprogramming [18]. We next replaced NEUROG2 with ASCL1, since both of them work as pioneer transcription factors dominantly controlling gene expression and neuronal fates [29, 30]. Furthermore, ASCL1^+^ progenitors can give rise to cholinergic neurons [23, 31–33].

Remarkably, a combination of the lentivirus *ASCL1-IRES-GFP-T2A*-*Sox11* and *LHX8-IRES-GBX1* (hereafter referred to as *ASLG*) enabled a majority (> 90%) of the virus-transduced adult NL fibroblasts (indicated by the co-expressed GFP) to become TUJ1^+^ and CHAT^+^ neuron-like cells at 28 dpi (Fig. 1b-d). During this conversion process, cells rapidly changed their initially flat, spread-out morphology to one with bipolar and multipolar processes. They exhibited round or pyramidal somas, condensed nuclei, long axons, and multiple neurites (Fig. 1b, Additional file 1: Figure S1A). Based on our prior experience with human induced motor neurons (hiMNs) [16, 18, 19], we also examined a polycistronic lentiviral vector, *LHX8-T2A-GBX1*, so that both LHX8 and GBX1 would be expressed roughly at an equal molar ratio. However, this vector caused mass cell loss when examined at 4 dpi (Additional file 1: Figure S1B) and produced very few neurons at 28 dpi (Additional file 1: Figure S1C, D).

Immunocytochemistry showed *ASLG*-induced neurons expressed markers for mature neurons such as neurofilament 200 (NF200), MAP2, and synapsin 1 (SYN1) when examined at 28 dpi (Fig. 1e, Additional file 2: Figure S2A). They also expressed neuronal cell adhesion molecule L1CAM (Additional file 2: Figure S2B). Importantly, more than 90% of GFP^+^ cells were stained positive for choline acetyltransferase (CHAT) (Fig. 1b-d) and vesicular acetylcholine transporter (VACHT) (Additional file 2: Figure S2C), two stereotypical markers for cholinergic neurons.

BFCNs are defined by their expression of neurotrophin receptor p75NTR and Trk receptors in addition to cholinergic markers [34–37]. In the basal forebrain p75NTR is colocalized exclusively with cholinergic neurons [38, 39]. Immunocytochemistry showed that the reprogrammed neurons expressed markers for BFCNs, including p75NTR and the transcription factor ISL1 (Fig. 1f, g, Additional file 2: Figure S2D). ISL1 is the earliest marker of cholinergic fate neurons and it forms complexes with LHX8 or LHX3 to enhance gene expression for cholinergic specification [40–42]. More than 95% GFP^+^ cells expressed ISL1 (Fig. 1c, g). On the other hand, these *ASLG*-induced neurons did not express HB9 (Fig.1h), an exclusive marker for cholinergic motor neurons as shown in hiMNs [18, 19] (Additional file 2: Figure S2E). Based on these above characteristics, we named the *ASLG*-induced neurons as human induced BFCNs (hiBFCNs). Fibroblasts from adult AD patients could be similarly reprogrammed by *ASLG* into hiBFCNs (Additional file 3: Figure S3).

The molecular properties of hiBFCNs were also examined by qRT-PCR (Fig. 1i). As controls, we included samples from human brains and fibroblast-converted hiMNs. hiBFCNs showed robust expression of genes enriched in neurons (*MAP2*, *MAPT*, *CALB1*) and BFCNs (*ISL1*, *NKX2.1*, *CHAT*, *VACHT*, *ACHE*, *TRKA*), whereas the motor neuron-specific marker *HB9* was not expressed. Due to contamination of non-converted fibroblasts in the samples, expression of fibroblast-enriched genes (*S100A4*, *VIM*) was detected but much reduced in hiBFCN samples.

### The electrophysiological properties of hiBFCNs

**Fig. 2:**
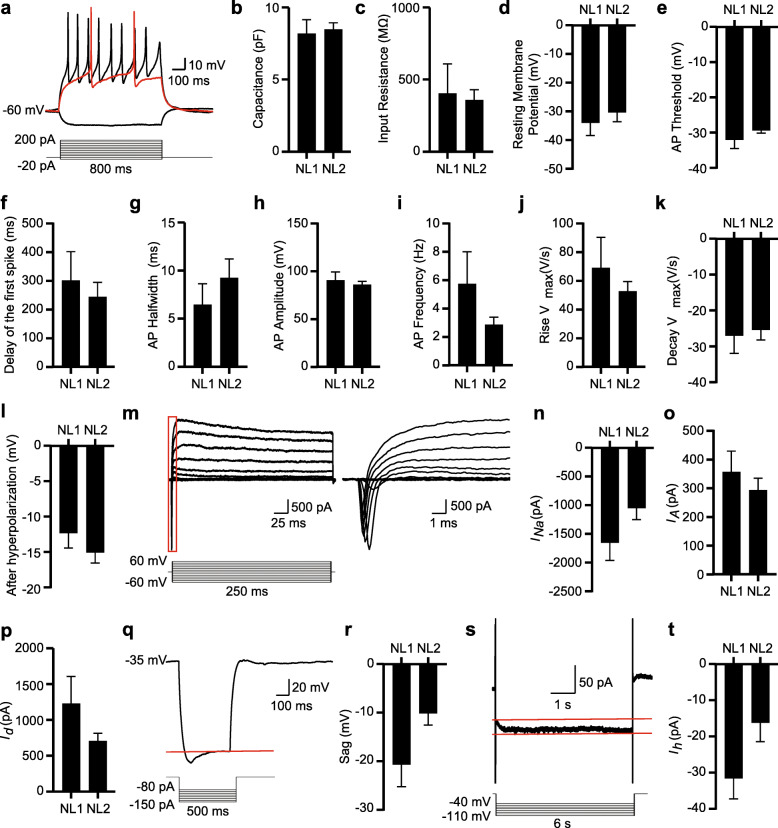
Electrophysiological properties of hiBFCNs. **a** Representative AP waveforms recorded under the current-clamp mode for a hiBFCN at 49 dpi. The black traces represent the precondition sweep and the sweep at the highest frequency. The red trace represents the sweep immediately above the threshold. A total of 44 out of 54 recorded cells fired APs. **b**-**d** Intrinsic properties of the recorded neurons from the indicated samples at between 49 and 55 dpi (mean ± SEM; *n* = 5 neurons for NL1, and *n* = 13 neurons for NL2). **e**-**l** Spiking characteristics of the APs for the indicated samples at between 49 and 55 dpi (mean ± SEM; *n* = 5 neurons for NL1, and *n* = 13 neurons for NL2). **m** Representative sodium and potassium currents under voltage-clamp mode for a recorded hiBFCNs at 55 dpi. An enlarged view of the boxed region is shown on the right. **n**-**p** Characteristics of ions currents for the recorded samples (mean ± SEM; *n* = 5 neurons for NL1, and *n* = 13 neurons for NL2). *I*_*Na*_, sodium current; *I*_*A*_, A-type potassium current; *I*_*d*_, delayed-rectifier potassium currents. **q** A representative voltage sag evoked by hyperpolarizing currents for a hiBFCN at 55 dpi. **r** Quantification of sag voltages for the recorded samples (mean ± SEM; *n* = 4 neurons for NL1, and *n* = 13 neurons for NL2). **s** Representative *I*_*h*_ evoked by hyperpolarizing voltage steps for a hiBFCN at 55 dpi. **t** Quantification of the *I*_*h*_ currents for the recorded samples (mean ± SEM; *n* = 4 neurons for NL1, and *n* = 12 neurons for NL2)

We used whole-cell patch-clamp recordings to examine the electrophysiological properties of hiBFCNs. A total of 44 out of 54 recorded hiBFCNs fired repetitive action potentials (APs) in response to depolarizing current injections in current clamp mode when examined at 49 dpi and beyond (Fig. 2a). Neuronal capacitance, input resistance, and resting membrane potentials were measured at 8.41 ± 0.40 pF, 372.0 ± 72.57 MΩ, and − 31.37 ± 2.60 mV, respectively (Fig. 2b-d). The AP threshold and delay of the first spike were − 30.14 ± 0.845 mV and 260.6 ± 44.68 ms, respectively (Fig. 2e, f). The AP half-width, amplitude, and frequency were approximately 8.48 ± 1.54 ms, 87.46 ± 3.27 mV, and 3.68 ± 0.75 Hz, respectively (Fig. 2g-i). On the other hand, the maximum velocity of rise and decay, and the after-hyperpolarization were 57.37 ± 7.48 V/s, − 25.87 ± 2.35 V/s and − 14.33 ± 1.21 mV, respectively (Fig. 2j-l).

In voltage-clamp mode, hiBFCNs showed typical inward sodium currents and outward potassium currents (Fig. 2m). The amplitudes of sodium currents (*I*_*Na*_), A-type potassium currents (*I*_*A*_), and delayed-rectifier potassium currents (*I*_*d*_) were respectively − 1221 ± 173.5 pA, 311.7 ± 35.36 pA, and 852.6 ± 135.9 pA (Fig. 2n-p). When injected with hyperpolarizing currents, voltage sags were observed in hiBFCNs (Fig. 2q, r). The hyperpolarization-activated currents (*I*_*h*_), elicited by hyperpolarizing voltage steps under voltage clamps, were about − 19.32 ± 4.54 pA (Fig. 2s, t). Together, these results show that hiBFCNs can become functionally mature exhibiting electrophysiological properties that are typical for mature neurons.

### hiBFCNs retain aging-associated features

**Fig. 3:**
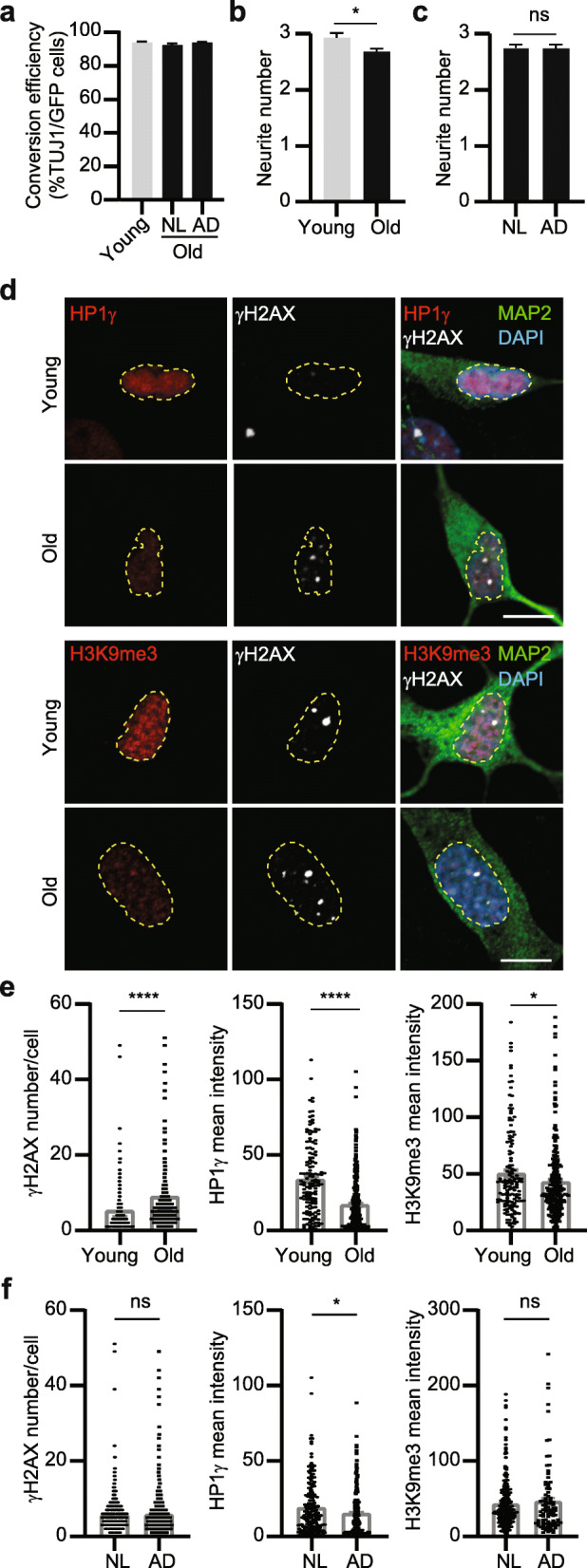
hiBFCNs retain aging-associated features. **a** Conversion efficiency for the indicated human fibroblast samples analyzed at 14 dpi. hiBFCNs were derived from young (2–3 years) and old (47–79 years) samples and co-cultured with mouse astrocytes (mean ± SEM; *n* = 3717 GFP^+^ cells for Young samples; *n* = 10,323 GFP^+^ cells for NL samples; and *n* = 8752 GFP^+^ cells for AD samples). **b** Quantification of primary neurite numbers per neuron for the indicated samples at 51 dpi (mean ± SEM; *n* = 361 cells for Young and *n* = 1052 for Old samples; *p = 0.0175). **c** Primary neurite numbers per neuron for the indicated samples at 51 dpi (mean ± SEM; *n* = 875 for NL and *n* = 538 for AD samples; ns, not significant). **d** Representative confocal images for marker expression in the indicated samples co-cultured with astrocytes at 51 dpi. The profiles of DAPI^+^ nuclei are outlined. Scale bar, 10 μm. **e** Quantifications of marker expression for the indicated young or old samples. Each dot represents a single cell (mean ± SEM; γH2AX: *n* = 426 for Young, *n* = 1263 for Old, ****p < 0.0001; HP1γ: *n* = 139 for Young, *n* = 421 for Old, ****p < 0.0001; H3K9me3: *n* = 146 for Young, *n* = 243 for Old; *p = 0.0446). **f** Quantifications of marker expression for the indicated NL or AD samples. Each dot represents a single cell (mean ± SEM; γH2AX: *n* = 621 for NL, *n* = 642 for AD; HP1γ: *n* = 200 for NL, *n* = 221 for AD, *p = 0.0162; H3K9me3: *n* = 243 for NL, *n* = 93 for AD; ns, not significant)

To examine whether directly reprogrammed hiBFCNs maintain aging-associated features, we derived hiBFCNs from fibroblasts of young (Young) and old (Old) human patients. The latter samples consisted of fibroblasts from both aged NL and sporadic AD patients. The reprogramming efficiency, which was about 92–94%, was similar between all these patient cells (Fig. 3a). Interestingly, hiBFCNs from old patients had markedly fewer primary neurites, despite no significant difference between NL- and AD-hiBFCNs when examined at 51 dpi (Fig. 3b, c).

We performed single-cell analysis after immunocytochemistry by using a set of molecular markers that were shown to reflect age-dependent cellular characteristics. These included γH2AX, trimethylated H3K9 (H3K9me3), and heterochromatin protein 1γ (HP1γ) [14, 16, 43]. hiBFCNs were co-cultured with astrocytes and analyzed at 51 dpi. Consistent with our previous results [16], hiBFCNs from older donors showed a much larger number of γH2AX foci than those from younger donors (*p* < 0.0001; Fig. 3d, e), whereas the expression level of H3K9me3 and HP1γ was significantly reduced in old than young hiBFCNs (*p* < 0.0001 for HP1γ and *p* = 0.0446 for H3K9me3; Fig. 3d, e). Very interestingly, HP1γ level was also markedly lower in AD than NL hiBFCNs (*p* = 0.0162; Fig. 3f), indicating that it could be a molecular marker for the diseased neurons. In contrast, hiBFCNs exhibited no disease-associated differences in terms of γH2AX foci per cell and the expression level of nuclear H3K9me3 (Fig. 3f). Together, these results indicate that hiBFCNs from older donors indeed retain certain aging-associated features, consistent with prior reports on other directly reprogrammed neurons from human fibroblasts [14–17].

### Relatively normal survival and soma size of AD-hiBFCNs

**Fig. 4:**
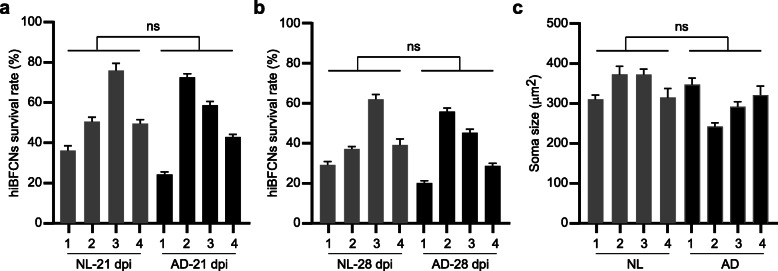
Cell survival and soma size. **a** Survival of the indicated hiBFCNs assayed at 21 dpi (mean ± SEM; ns, not significant). **b** Survival of the indicated hiBFCNs assayed at 28 dpi (mean ± SEM; ns, not significant). **c** Quantification of soma size for the indicated hiBFCNs at 51 dpi (mean ± SEM; *n* = 192 for NL1, *n* = 134 for NL2, *n* = 216 for NL3, *n* = 82 for NL4, *n* = 178 for AD1, *n* = 180 for AD2, *n* = 220 for AD3, and *n* = 69 for AD4; ns, not significant)

The survival of hiBFCNs was determined in co-culture with wild-type mouse astrocytes, which were required in general for neuronal growth and long-term culture. After replating at 14 dpi, survived hiBFCNs were quantified at 21 and 28 dpi. Cell counts were then normalized to the starting neuronal number for each sample at 14 dpi. The survival rates were heterogeneous among all the human samples, ranging from about 24 to 76% at 21 dpi and 20 to 62% at 28 dpi (Fig. 4a, b). Statistical analysis failed to show a significant difference between NL- and AD-hiBFCNs at both time points. Similarly, both NL- and AD-hiBFCNs showed heterogeneous but not significantly different soma sizes when analyzed at 51 dpi (242–373 μm^2^; Fig. 4c). These results indicate that AD-hiBFCNs do not exhibit intrinsic deficits on cell survival or soma sizes.

### Aging-associated TAU hyperphosphorylation in AD-hiBFCNs

**Fig. 5:**
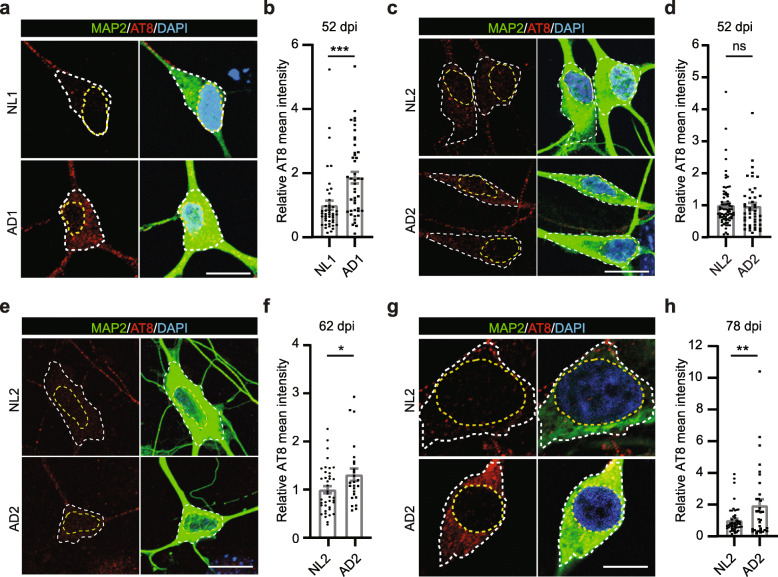
Time-dependent TAU hyperphosphorylation. **a** Representative confocal images of marker expression in the indicated hiBFCNs at 52 dpi. Cell soma and nucleus are outlined, respectively. Scale bar, 10 μm. **b** Quantification of soma AT8 expression in the indicated hiBFCNs at 52 dpi. The dots indicate single cells (mean ± SEM; *n* = 40 for NL1; *n* = 41 for AD1; ***p = 0.0004). **c** Representative confocal images of marker expression in the indicated hiBFCNs at 52 dpi. Scale bar, 10 μm. **d** Quantification of soma AT8 expression in the indicated hiBFCNs at 52 dpi (mean ± SEM; *n* = 66 for NL2; *n* = 41 for AD2; ns, not significant). **e** Representative confocal images of marker expression in the indicated hiBFCNs at 62 dpi. Scale bar, 10 μm. **f** Quantification of soma AT8 expression in the indicated hiBFCNs at 62 dpi (mean ± SEM; *n* = 39 for NL2; *n* = 25 for AD2; *p = 0.0298). **g** Representative confocal images of marker expression in the indicated hiBFCNs at 78 dpi. Scale bar, 10 μm. **h** Quantification of soma AT8 expression in the indicated hiBFCNs at 78 dpi (mean ± SEM; *n* = 50 for NL2; *n* = 30 for AD2; **p = 0.0078)

AD-related tauopathy arises early in BFCNs and parallels cognitive decline [44, 45]. We examined phosphorylated TAU through western blotting and immunocytochemistry by using the well-established AT8 antibody [46–50]. We used age- and gender-matched sample pairs cultured at the same time to reduce the potential influence of biological variabilities on phenotype. hiBFCNs were co-cultured with primary mouse astrocytes. When examined by western blotting at 28 dpi, no marked difference was observed for AT8 expression in NL- and AD-hiBFCNs (Additional file 4: Figure S4A). Since we failed to obtain enough hiBFCNs for western blotting at later culture time points, we focused our analysis on immunohistochemistry. When examined at 52 dpi and compared to the control NL1-hiBFCNs (70 YR, female, *APOE3/3*), AD1-hiBFCNs (62 YR, female, *APOE3/4*) showed much elevated hyperphosphorylated TAU in the somas (*p* = 0.0004; Fig. 5a, b). Interestingly, this phenotype was delayed when comparing to another sample pair from younger individuals, NL2-hiBFCNs (47 YR, male, *APOE3/3*) and AD2-hiBFCNs (47 YR, male, *APOE3/4*). The increased hyperphosphorylated TAU phenotype in AD2-hiBFCNs was not observed at the early time point 52 dpi (Fig. 5c, d); but it was evident at 62 dpi and became even more significant at 78 dpi (*p* = 0.0298 for 62 dpi and *p* = 0.0078 for 78 dpi; Fig. 5e-h). In contrast to the dysregulated TAU phosphorylation in somas, neuritic AT8 expression was similar in NL- and AD-hiBFCNs (Additional file 4: Figure S4B, C).

### Time-dependent impairment of nucleocytoplasmic transport in AD-hiBFCNs

**Fig. 6:**
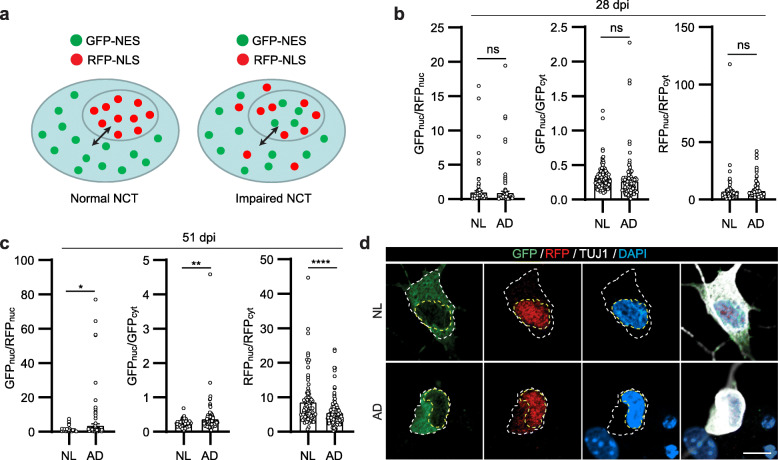
Time-dependent impairment of nucleocytoplasmic transport activity in hiBFCNs. **a** A schematic of the reporter system for analyzing NCT activity. **b** Quantification of subcellular distributions of the reporters at 28 dpi. Each point represents a single cell (mean ± SEM; *n* = 124 for NL from 4 samples; *n* = 145 for AD from 4 samples; ns, not significant). **c** Quantification of subcellular distributions of the reporters at 51 dpi. Each point represents a single cell (mean ± SEM; *n* = 92 for NL from 3 samples; *n* = 127 for AD from 4 samples; *p = 0.0309, **p = 0.0067, and ****p < 0.0001). **d** Representative confocal images of reporter subcellular distributions in hiBFCNs at 51 dpi. Cell soma and nucleus are outlined. Scale bar, 10 μm

TAU hyperphosphoration leads to disrupted nucleocytoplasmic transport (NCT) in AD neurons [51]. We examined NCT activity in hiBFCNs by using a well-established reporter assay [15, 51, 52]. This reporter (2Gi2R) consists of 2xGFP containing an NES sequence (GFP-NES), an internal ribosome entry site (IRES), followed by 2xRFP containing an NLS sequence (RFP-NLS). GFP and RFP are localized in the cytoplasm and nucleus, respectively, in cells with normal NCT activity; however, such subcellular distribution of reporters will be disrupted in cells with abnormal NCT (Fig. 6a). A higher ratio of nuclear GFP to RFP (GFP_nuc_/RFP_nuc_) represents disrupted NCT, whereas the nuclear to cytoplasmic GFP (GFP_nuc_/GFP_cyt_) shows impaired protein export and a lower ratio of the nuclear to cytoplasmic RFP (RFP_nuc_/RFP_cyt_) indicates compromised protein nuclear import.

The 2Gi2R reporter was introduced into hiBFCNs during the initial reprogramming process. Neurons were co-cultured with primary astrocytes until analysis by immunocytochemistry. Fluorescence intensity of the reporters in individual neurons was respectively measured in the nucleus or cytoplasm based on DAPI staining from confocal image sections. While no significant differences were observed between NL- and AD-hiBFCNs at the early 28 dpi (Fig. 6b), AD-hiBFCNs showed markedly increased ratios of GFP_nuc_/RFP_nuc_ or GFP_nuc_/GFP_cyt_ when compared to the control NL group (*p* = 0.0309 for GFP_nuc_/RFP_nuc_ and *p* = 0.0067 for GFP_nuc_/GFP_cyt_; Fig. 6c, d). Conversely, the RFP_nuc_/RFP_cyt_ ratios were significantly decreased in AD-hiBFCNs than the controls (*p* < 0.0001 for RFP_nuc_/RFP_cyt_; Fig. 6c, d).To examine whether such dysregulated reporter distribution might be due to nuclear membrane breakdown, we treated 2Gi2R-expressing hiBFCNs with leptomycin B (LMB), a potent and specific inhibitor of nuclear exports. Time-lapse live-cell confocal imaging and immunocytochemistry showed that both NL- and AD-hiBFCNs robustly responded to LMB treatments, indicating that these cells had functional nuclear membranes (Additional file 5: Figure S5A-E). Together, these results indicate that AD-hiBFCNs exhibit time-dependent impairments of NCT activities.

## Discussion

BFCNs critically regulate brain function through projections to the cortex, hippocampus, and thalamus [1]. Their dysfunction is an early hallmark of AD [2, 3, 53, 54]. Our direct induction of hiBFCNs from human patient fibroblasts provides a much-needed cell model to understand their molecular and cellular pathology in AD. These neurons retain certain aging-associated features that are critical to understating adult-onset neurodegenerative AD. Our proof-of-concept study indeed reveals some potential defects in hiBFCNs from AD patients. These include time-dependent TAU hyperphosphorylation and dysfunctional nucleocytoplasmic transport. Such preliminary results warrant future studies with additional patient samples. hiBFCNs especially those from AD patients may also be employed to screen or validate small molecules as therapeutics for AD.

The replacement of NEUROG2 with ASCL1 is critical for hiBFCNs, although both of them can work as pioneer factors during neuronal reprogramming of fibroblasts [55, 56]. We previously demonstrated that human skin fibroblasts can be directly and efficiently converted into cholinergic neurons by the combined actions of NEUROG2, SOX11, and small molecules [18]. During the reprogramming process, NEUROG2 acts as a pioneer factor, whereas SOX11 facilitate fate transition and promote neuronal survival and maturation [18, 55]. Approximately 80% of the NEUROG2 and SOX11-induced neurons are motor neuron-like with unique early expression of HB9, a key transcription factor restricted to spinal motor neurons [18]. These neurons can be further coerced into hiMNs with the inclusion of ISL1 and LHX3, two transcription factors critical for motor neuron development [57]. However, when we combined NEUROG2 and SOX11 with LHX8 and GBX1, two factors essential for BFCNs [7, 12, 22–26], HB9^+^ motor neuron-like cells were still observed. Such a result clearly shows a dominant role of NEUROG2 in motor neuron reprogramming. On the other hand, replacing NEUROG2 with ASCL1 completely eliminated generation of HB9^+^ cells and produced hiBFCNs.

Without passing through a pluripotent stem cell state, the direct reprogramming process for hiBFCNs is rapid and efficient. When examined at 28 dpi, more than 90% of those virus-transduced cells become neurons expressing stereotypical markers for BFCNs, such as CHAT, p75NTR, ISL1, and VACHT. qRT-PCR results further confirmed BFCN lineage. hiBFCNs become mature at 49 dpi and beyond, showing typical inward sodium currents and outward potassium currents and firing repetitive APs when stimulated. They also show higher expression of L1CAM and exhibit more mature cell morphology at 78 dpi.

During early stage of the reprogramming process, cell death is a main reason causing neuronal loss. Cell death could be caused through apoptosis, necroptosis, and other pathways [58, 59]. The replating step at 14 dpi, which is important for partial purification of the converted neurons, may also result in axotomy and subsequent axonal degeneration and cell death [59]. Interestingly, both NL- and AD-hiBFCNs respond similarly to the reprogramming process and the replating procedure, as we do not detect significant differences of cell survival or soma size before 28 dpi. Nonetheless, the reprogramming procedure may be further optimized for higher neuronal yield and purity in the future. Fibroblasts from different human patients also exhibit heterogeneity, which may lead to variable virus-transduction efficiency, cell survival, and neuronal yield.

We and others have recently demonstrated that directly induced neurons from adult human fibroblasts retain aging-associated signatures, which could be erased if passing through a pluripotent stem cell stage [15–17]. Some of these signatures include age-specific transcriptional and epigenetic profiles and age-dependent changes on DNA damage, chromatin structure, nuclear organization, and nucleocytoplasmic compartmentalization. Retaining these aging-associated features is critically important for understanding late-onset neurodegeneration such as AD, since advanced age is the greatest known risk factor. Consistent with other directly induced neurons [14–17], our hiBFCNs also retain certain aging-associated signatures as their parental fibroblasts.

A disadvantage of directly induced neurons is their limited number and heterogeneity. Unlike ESCs or iPSCs which possess self-renewal ability, adult human fibroblasts become senescent after a limited number of passages. Directly induced neurons including hiBFCNs will be better suited for single cell analyses, such as immunocytochemistry, electrophysiology, and single-cell transcriptomics and genomics. Because long-term survival also requires co-culture with healthy astrocytes, hiBFCNs and some other directly converted neurons from adult human fibroblasts may not be well suited for biochemical analysis such as western and northern blotting. Directly induced neurons including hiBFCNs are also heterogeneous, since their parental fibroblasts cannot be single-cell cloned and be made isogenic through gene-editing. On the other hand, such heterogeneity may well resemble endogenous conditions in human patients. A good practice is to use age- and gender-matched sample pairs, as we have done in this proof-of-concept study.

## Conclusions

We established a protocol for the generation of aging relevant BFCNs from adult human skin fibroblasts including those of sporadic AD patients. To our knowledge, this is the first study in which direct lineage reprogramming bypasses pluripotency and converts fully differentiated adult somatic cells into electrophysiologically mature hiBFCNs. Our proof-of-concept study further reveals that hiBFCNs show promises as a cell model for understanding AD pathology, including tauopathy and nucleocytoplasmic transport dysfunction. The availability of these cells may also facilitate therapeutics identification for AD patients.

## Methods

### Animals

Wild-type C57BL/6 J mice were obtained from The Jackson Laboratory. All mice were housed under a controlled temperature and 12-h light/dark cycle with ad libitum access to food and water in the UT Southwestern animal facility. All experimental procedures and protocols were approved by the Institutional Animal Care and Use Committee at UT Southwestern.

### Plasmid construction and lentivirus preparation

A third-generation, replication-deficient lentiviral vector (*pCSC-SP-PW-IRES-GFP*) was used to express *NEUROG2-IRES-GFP-T2A-Sox11*, *ASCL1-IRES-GFP-T2A-Sox11*, *ASCL1-IRES-Sox11*, *ISL1-T2A-LHX3*, *LHX8-T2A-GBX1*, *LHX8-IRES-GBX1*, *ASCL1-P2A-NEUROG2-IRES-Sox11*. HEK293 cells (ATCC) were maintained in 10% FBS DMEM medium (with 1% penicillin/streptomycin) with 5% CO_2_ at 37 °C. HEK293 cells were seeded at 3 × 10^6^ cells in 10 ml medium per 10-cm dish 24 h before transfection. Lentiviruses were generated in HEK293 cells by transient transfections with lentiviral vectors and the packaging plasmids (*pMDL*, *VSV-G* and *pREV*) at a mass ratio of 4:2:1:0.5 (3 μl PEI/μg plasmids) for third-generation lentiviral vector (at 4 μg: 2 μg: 1 μg: 0.5 μg with 22.5 μl PEI in 1 ml DMEM for 10-cm dish). The *2Gi2R* lentivirus was generated in HEK293 cells by transient transfections with lenti *XEP.2GFP:NES-IRES-2RFP:NLS* and the packaging plasmids (*psPAX2* and *VSV-G*) at a mass ratio of 2:2:1 (3 μl PEI/μg plasmids) (at 10 μg: 10 μg: 5 μg with 75 μl PEI in 1 ml DMEM for 10-cm dish). The mixture was incubated for 15 min at RT before adding to the culture. Medium was refreshed after 12 h to 15 h incubation. Viruses were collected at 24 h and refreshed with new medium again for one more collection at 48 h. These two collections were combined. Lentiviral supernatants were cleared with 0.45 μm filter and stored at 4 °C prior to cell transduction.

### Human fibroblast culture

All human fibroblasts from either healthy controls or sporadic AD patients with different *APOE* alleles were purchased from the Coriell Institute for Medical Research (Table 1). They were maintained in Fibroblast Medium (DMEM containing 15% FBS and 1% penicillin/streptomycin) with 5% CO_2_ at 37 °C.

**Table 1:** Human fibroblasts used in this study

Sample Name	Company	Catalog ID	Origin	Age	Gender	Disease	APOE isoforms
Y-1	Coriell	GM00969	Skin	2 Years	Female	Apparently Healthy	–
Y-2	Coriell	GM05565	Skin	3 Years	Male	Apparently Healthy	–
NL1	Coriell	AG11733	Skin	70 Years	Female	Apparently Healthy	*ɛ 3/3*
NL2	Coriell	AG12989	Skin	47 Years	Male	Apparently Healthy	*ɛ 3/3*
NL3	Coriell	AG07479	Skin	70 Years	Female	Apparently Healthy	*–*
NL4	Coriell	AG12934	Skin	77 Years	Male	Apparently Healthy	*ɛ 3/3*
AD1	Coriell	AG06264	Skin	62 Years	Female	AD	*ɛ 3/4*
AD2	Coriell	AG04402	Skin	47 Years	Male	AD	*ɛ 3/4*
AD3	Coriell	AG21158	Skin	69 Years	Female	AD	*ɛ 2/3*
AD4	Coriell	AG05810	Skin	79 Years	Male	AD	*ɛ 3/4*

### Neuron induction and culture

Direct lineage reprogramming was conducted according to a previous protocol with some modifications [18, 19]. In brief, fibroblasts were seeded onto Matrigel-coated culture vessels (4.8 × 10^5^ cells per 24- or 48-well plate or 3 × 10^5^ cells per 6-cm dish or 1 × 10^6^ cells per 10-cm dish) and cultured in Fibroblast Medium for 1 day. Then, cells were transduced with lentiviral supernatants in the presence of 8 μg/ml polybrene. Fibroblast culture medium was refreshed after overnight incubation. One day later, these cells were switched into Reprogramming Medium (C2 medium supplemented with 10 μM FSK (Sigma-Aldrich), 1 μM LDN-193189 (EMD Millipore), and 10 ng/ml FGF2 (PeproTech)). The C2 medium was composed of DMEM:F12:neurobasal (1:1:1), 0.8% N2 (Invitrogen), 0.8% B27 (Invitrogen), and 1% penicillin/streptomycin. The Reprogramming Medium was half changed every other day until 14 dpi. These cells were dissociated with 0.05% trypsin for 3 min at 37 °C and resuspended in Fibroblast Medium to quench trypsin activity. This cell suspension was then plated into a 0.1% gelatin-coated culture dish on which contaminating fibroblasts could tightly attach. About one and a half hours later, floating cells, which mainly consisted of induced neurons, were collected by centrifugation at 400 g for 3 min. Cells were resuspended into C2 medium and centrifuged again to remove cell debris. Finally, induced neurons were plated into primary astrocytes-coated plates in Maturation Medium (C2 medium supplemented with 5 μM FSK, 10 ng/ml each of BDNF, GDNF, and NT3 (PeproTech), 50 ng/ml NGFβ (PeproTech)). Unless indicated otherwise, Maturation Medium was half changed twice a week. For conversion efficiency calculation, induced neurons were plated into primary astrocytes-coated 96-well plates (at least three wells per condition). Fourteen days after replating (at 28 dpi) in maturation medium, cells were fixed and stained with antibodies for GFP, TUJ1, CHAT, or ISL1. Nuclei were counterstained with DAPI. The percentage of TUJ1^+^GFP^+^ cells among total GFP^+^ cells was calculated as conversion efficiency. The percentage of CHAT^+^TUJ1^+^ among TUJ1^+^ cells was calculated as conversion purity. The percentage of CHAT^+^ or ISL1^+^ cells among total GFP^+^ cells was also calculated. Human induced motor neurons (hiMNs) were generated essentially as described previously [19].

### Immunocytochemistry

Cell cultures at the indicated time points were fixed with 4% paraformaldehyde (PFA) in PBS for 15 min at room temperature, twice-washed with PBS, and then permeabilized/blocked for 1 h in blocking solution (1 x PBS containing 0.2% Triton X-100 and 3% BSA). Primary antibodies (Table S1) in blocking solution were then added and incubated overnight at 4 °C, followed by PBS washing and incubation with Alexa Fluor-conjugated corresponding secondary antibodies made in donkey (Invitrogen, 1:500). Images were obtained with a NIKON A1R confocal microscope. The mean fluorescence intensity of AT8 staining in the cytosol or neurites of each neuron was quantified using Image J.

### Cell survival analysis

hiBFCNs co-cultured with primary mouse astrocytes were used for survival analysis. Cortical astrocytes were prepared as previously described with modifications [20]. Briefly, cortices were dissociated with a solution containing papain (10 U/ml, with 1 mM Ca^2+^ and 0.5 mM EDTA) and 1% DNase for 20 min at 37 °C. Tissues were pelleted through brief centrifugation and further dissociated using a pipette in FBS-containing medium. Cells were passed through a 40 μm nylon strainer. The cell mixture was spun at 400 g for 3 min and re-suspended in growth media consisting of DMEM (Invitrogen) supplemented with 10% FBS and plated into 0.1% gelatin coated 75 cm^2^ flasks. Media was exchanged every 3 days. Endogenous mouse neurons and non-astrocytes were removed by vigorous shaking and a few cycles of passaging, freezing, thawing, and replating. hiBFCNs at 14 dpi were replated into astrocyte-coated 96-well plates (survival analysis) or coverslip-containing 24-well plates (for neuronal morphology analysis). Co-cultures were fed twice a week with Maturation Medium. Under an AMG EVOS digital inverted fluorescence microscope, TUJ^+^GFP^+^ cells within the entire well of a 96-well plate in triplicate were quantified by a researcher blinded to experimental groups. Cell counts were normalized to the starting number of cells plated into each well. Survival rate at 14 dpi for each cell line was set as 100%, to which the subsequent survived cells at each time point were normalized.

### Quantitative RT-PCR (qRT-PCR)

hiBFCNs and hiMNs were cultured in Matrigel-coated plates until the indicated time point. They were then resuspended through gentle pipetting with 0.5 ~ 1 ml culture medium to partially remove contaminating fibroblasts. Cells were pelleted by 800 g × 5 min at 4 °C and lysed in TRIzol reagent (Invitrogen). Total RNA was isolated by using a commercial kit (RNA Clean & Concentrator kits, ZYMO Research). Human brain total RNA was purchased from Clontech (Cat#: 636530). cDNA was synthesized from total RNA (50 ~ 100 ng per sample) with the SuperScriptIII First-Strand Synthesis kit (Invitrogen). Real-Time PCR was performed with the SYBR GreenER SuperMix (Invitrogen) on the QuantStudio 5 Real-Time PCR System (Thermo Fisher). Primer sequences are listed in Supplementary Table S2 and their quality was assessed by the dissociation curve. Relative gene expression was determined by using the 2^-ΔΔCt^ method after normalization to the loading control *GAPDH*.

### Nucleocytoplasmic transport

The 2Gi2R reporter-expressing lentivirus was included during the reprogramming of fibroblasts to hiBFCNs. Cells were replated onto astrocyte-coated and Matrigel-treated coverslips. At the indicated time points, cells were fixed with 4% PFA, followed by immunostaining with antibodies against GFP, RFP, and TUJ1. Nuclei were counterstained with DAPI. For live-cell imaging, cells at the indicated time point were maintained at 37 °C with 5% CO_2_ under the Nikon A1R confocal microscope system. The target cells were located under a 60x objective and imaged as 0 min. Culture medium was then replaced with prewarmed medium containing 50 nM leptomycin B (LMB). Cells were subsequently imaged every 10 min for a total of 1 h, followed by fixation with 4% PFA and immunocytochemistry. Images of single confocal plane across the center of the nucleus were obtained on the NIKON A1R confocal microscope with a pinhole setting at 2.5. Because of the complexity of neuron-astrocyte co-cultures, neuronal nucleus and soma were manually defined by using the Image J program. The mean fluorescence intensity of GFP or RFP was separately measured in the cytoplasm or nucleus of hiBFCNs. The ratios of GFP_nuc_/RFP_nuc_, GFP_nuc_/GFP_cyt_ and RFP_nuc_/RFP_cyt_ were calculated by Microsoft Excel and analyzed by GraphPad Prism 8.

### Western blotting

hiBFCNs were co-cultured with astrocytes in Matrigel-coated plates. As controls, generic human induced neurons (hiNs) were produced through reprogramming adult skin fibroblasts with the lentivirus expressing *ASCL1-P2A-NEUROG2-IRES-Sox11*. Reprogramming Medium (C2 medium supplemented with 5 μM FSK (Sigma-Aldrich), 1 μM LDN-193189 (EMD Millipore), 1 μM Dorsomorphin (EMD Millipore), 0.5 μM A83–1 (Tocris), 3 μM CHIR99021 (Selleckchem), 10 μM SB-431542 (Selleckchem), 1 μg/ml laminin (Corning) and 10 ng/ml FGF2 (PeproTech)) was used for hiNs induction. hiNs were replated into Matrigel-coated plates at 16 dpi. Cells were maintained in Maturation Medium (C2 medium supplemented with 5 μM FSK, 10 ng/ml each of BDNF, GDNF, and NT3 (PeproTech)). At the indicated time points, cells were collected and lysed in buffer composed of 50 mM Tris-HCl buffer (pH 7.5), 150 mM NaCl, 1% Triton X-100, 0.1% SDS, 0.5% sodium deoxycholate, protease inhibitors (Pierce), and PhosSTOP (Roche). Protein concentration was measured by the Bradford assay. Protein samples were separated by 12% SDS-PAGE and transferred to PVDF membranes. After blocking in 5% nonfat milk in PBST for 1 h at room temperature, membranes were incubated overnight with primary antibodies at 4 °C. Horseradish peroxidase-conjugated secondary antibodies (Jackson) were applied, and the blots were developed with Pierce ECL Western Blotting Substrate (Thermo-Fisher) or Immobilon Western Chemiluminescent HRP substrate (Millipore-Sigma). Antibodies are listed in Table S1.

### Electrophysiology

Whole-cell patch-clamp recordings were made under visual guidance using infra-red differential interference contrast (IR-DIC) and GFP fluorescence to identify GFP^+^ cells. For analysis of intrinsic neuronal properties, cells were maintained at 30 °C in a submersion chamber with Tyrode solution containing 150 mM NaCl, 4 mM KCl, 2 mM MgCl_2_, 3 mM CaCl_2_, 10 mM glucose, and 10 mM HEPES at pH 7.4 (adjusted with KOH) and 300 mOsm. Whole-cell recordings were performed on induced neurons using recording pipettes (approximately 5–9 MΩ) filled with intracellular solution (0.2 mM EGTA, 130 mM K-Gluconate, 6 mM KCl, 3 mM NaCl, 10 mM HEPES, 4 mM ATG-Mg, 0.4 mM GTP-Na, 14 mM phosphocreatine-di(Tris) at pH 7.2 (adjusted by KOH) and 285 mOsm). Series and input resistance were measured in voltage-clamp mode with a 400 ms, 10 mV step from a − 60 mV holding potential (filtered at 10 kHz, sampled at 50 kHz). Cells were only accepted for analysis if the series resistance was less than 30 MΩ and stable (< 10% change) throughout the experiment. Input resistance ranged from 0.2 to 2 GΩ. Currents were filtered at 3 kHz, acquired and digitized at 10 kHz on a PC using Clampex10.3 software (Molecular Devices). A MultiClamp 700B amplifier (Molecular Devices, Palo Alto, CA) was used for recordings.

Action potentials were recorded in current clamp mode and elicited by a series of current injections starting from − 20 to 200 pA with 20-pA increments and 800-ms in duration. Sodium and potassium currents were recorded in voltage-clamp mode in response to a series of voltage steps ranging from − 60 to + 60 mV at 10-mV increments and 250-ms duration according to standard protocols. Sag voltage was recorded in current-clamp mode with hyperpolarizing current (− 80 to − 150 pA, 500 ms**)**. *I*_*h*_ was recorded in voltage-clamp mode by injecting voltage steps from − 110 to − 40 mV with 10-mV increments for 6 s duration, and an average of 10 traces. In all voltage-clamp recordings, cells were clamped at − 60 mV except during the voltage-step protocol. In all current-clamp recordings, recordings were made at resting membrane potential or without any current injection except otherwise stated.

Data analysis was performed using Clamp-fit 10.3 software (Molecular Devices). The action potential (AP) was analyzed as described previously [19]. The AP trace immediately above threshold was used to determine the delay of 1st spike as the length of time from the start of current steps to the peak of AP. The same AP trace was used to measure AP threshold as the corresponding voltage when there was the sharpest change of trace slope. The above-indicated AP trace was also used to determine AP amplitude, halfwidth, maximum velocity of rise, and decay slope using the “Statistics” function from the “Analyze” menu. AP frequency was obtained by dividing the maximum number of spikes during the current steps protocol with the step time duration (800 ms). Similarly, sodium and potassium currents were measured using the “Statistics” function. The biggest current was used.

### Statistical analysis

All experiments were performed at least twice in triplicate unless otherwise indicated. Data were presented as mean ± SEM. One-way ANOVA or unpaired Student’s t-test was used to calculate statistical significance in GraphPad Prism. Significant differences are indicated by *p < 0.05, **p < 0.01, ***p < 0.001, and ****p < 0.0001.

## Supplementary information

**Additional file 1: Figure S1**. The reprogramming process and optimization, related to Fig. 1 A.

**Additional file 2: Figure S2**. Characterization of the induced neurons, related to Fig. 1 A.

**Additional file 3: Figure S3.** Characterization of AD-hiBFCNs, related to Fig. 1 A.

**Additional file 4: Figure S4.** PhosphoTAU expression in hiBFCNs, related to Fig. 5 A.

**Additional file 5: Figure S5.** Effect of LMB on nucleocytoplasmic transport in hiBFCNs, related to Fig. 6 A.

**Additional file 6: Table S1.** Primary antibodies. **Table S2.** Primer sequences.

## Data Availability

All data generated or analyzed during this study are included in the article and its supplementary information files.

## Abbreviations

AD: Alzheimer’s disease
APs: Action potentials
APOE: Apolipoprotein E
BFCNs: Basal forebrain cholinergic neurons
CHAT: Choline acetyltransferase
FGF2: Fibroblast growth factor beta
FSK: Forskolin
*I*_*A*_: A-type potassium current
*I*_*d*_: Delayed-rectifier potassium current
*I*_*h*_: Hyperpolarization-activated current
*I*_*Na*_: Sodium current
MNs: Motor neurons
NL: Normal line
NF200: Neurofilament 200
qRT-PCR: Quantitative RT-PCR
SYN1: Synapsin 1
VACHT: Vesicular acetylcholine transporter

## References

[1] Mesulam MM, Mufson EJ, Wainer BH, Levey AI (1983). Central cholinergic pathways in the rat: an overview based on an alternative nomenclature (Ch1-Ch6). Neuroscience.

[2] Whitehouse PJ, Price DL, Struble RG, Clark AW, Coyle JT, Delon MR (1982). Alzheimer's disease and senile dementia: loss of neurons in the basal forebrain. Science.

[3] Coyle JT, Price DL, DeLong MR (1983). Alzheimer's disease: a disorder of cortical cholinergic innervation. Science.

[4] Yue W, Li Y, Zhang T, Jiang M, Qian Y, Zhang M, Sheng N, Feng S, Tang K, Yu X (2015). ESC-derived basal forebrain cholinergic neurons ameliorate the cognitive symptoms associated with Alzheimer's disease in mouse models. Stem Cell Reports.

[5] Liu Y, Weick JP, Liu H, Krencik R, Zhang X, Ma L, Zhou GM, Ayala M, Zhang SC (2013). Medial ganglionic eminence-like cells derived from human embryonic stem cells correct learning and memory deficits. Nat Biotechnol.

[6] Kwakowsky A, Potapov K, Kim S, Peppercorn K, Tate WP, Abraham IM (2016). Treatment of beta amyloid 1-42 (Abeta(1-42))-induced basal forebrain cholinergic damage by a non-classical estrogen signaling activator in vivo. Sci Rep.

[7] Bissonnette CJ, Lyass L, Bhattacharyya BJ, Belmadani A, Miller RJ, Kessler JA (2011). The controlled generation of functional basal forebrain cholinergic neurons from human embryonic stem cells. Stem Cells.

[8] Wicklund L, Leao RN, Stromberg AM, Mousavi M, Hovatta O, Nordberg A, Marutle A (2010). Beta-amyloid 1-42 oligomers impair function of human embryonic stem cell-derived forebrain cholinergic neurons. PLoS One.

[9] Moreno CL, Della Guardia L, Shnyder V, Ortiz-Virumbrales M, Kruglikov I, Zhang B, Schadt EE, Tanzi RE, Noggle S, Buettner C, Gandy S (2018). iPSC-derived familial Alzheimer's PSEN2 (N141I) cholinergic neurons exhibit mutation-dependent molecular pathology corrected by insulin signaling. Mol Neurodegener.

[10] Hu Y, Qu ZY, Cao SY, Li Q, Ma L, Krencik R, Xu M, Liu Y (2016). Directed differentiation of basal forebrain cholinergic neurons from human pluripotent stem cells. J Neurosci Methods.

[11] Duan L, Bhattacharyya BJ, Belmadani A, Pan L, Miller RJ, Kessler JA (2014). Stem cell derived basal forebrain cholinergic neurons from Alzheimer's disease patients are more susceptible to cell death. Mol Neurodegener.

[12] Crompton LA, Byrne ML, Taylor H, Kerrigan TL, Bru-Mercier G, Badger JL, Barbuti PA, Jo J, Tyler SJ, Allen SJ (2013). Stepwise, non-adherent differentiation of human pluripotent stem cells to generate basal forebrain cholinergic neurons via hedgehog signaling. Stem Cell Res.

[13] Ortiz-Virumbrales M, Moreno CL, Kruglikov I, Marazuela P, Sproul A, Jacob S, Zimmer M, Paull D, Zhang B, Schadt EE (2017). CRISPR/Cas9-correctable mutation-related molecular and physiological phenotypes in iPSC-derived Alzheimer's PSEN2 (N141I) neurons. Acta Neuropathol Commun.

[14] Mertens J, Reid D, Lau S, Kim Y, Gage FH (2018). Aging in a dish: iPSC-derived and directly induced neurons for studying brain aging and age-related neurodegenerative diseases. Annu Rev Genet.

[15] Mertens J, Paquola ACM, Ku M, Hatch E, Bohnke L, Ladjevardi S, McGrath S, Campbell B, Lee H, Herdy JR (2015). Directly reprogrammed human neurons retain aging-associated Transcriptomic signatures and reveal age-related Nucleocytoplasmic defects. Cell Stem Cell.

[16] Tang Y, Liu ML, Zang T, Zhang CL (2017). Direct reprogramming rather than iPSC-based reprogramming maintains aging hallmarks in human motor neurons. Front Mol Neurosci.

[17] Huh CJ, Zhang B, Victor MB, Dahiya S, Batista LF, Horvath S, Yoo AS (2016). Maintenance of age in human neurons generated by microRNA-based neuronal conversion of fibroblasts. Elife.

[18] Liu ML, Zang T, Zou Y, Chang JC, Gibson JR, Huber KM, Zhang CL (2013). Small molecules enable neurogenin 2 to efficiently convert human fibroblasts into cholinergic neurons. Nat Commun.

[19] Liu ML, Zang T, Zhang CL (2016). Direct lineage reprogramming reveals disease-specific phenotypes of motor neurons from human ALS patients. Cell Rep.

[20] Vierbuchen T, Ostermeier A, Pang ZP, Kokubu Y, Sudhof TC, Wernig M (2010). Direct conversion of fibroblasts to functional neurons by defined factors. Nature.

[21] Matsumoto K, Tanaka T, Furuyama T, Kashihara Y, Mori T, Ishii N, Kitanaka J, Takemura M, Tohyama M, Wanaka A (1996). L3, a novel murine LIM-homeodomain transcription factor expressed in the ventral telencephalon and the mesenchyme surrounding the oral cavity. Neurosci Lett.

[22] Mori T, Yuxing Z, Takaki H, Takeuchi M, Iseki K, Hagino S, Kitanaka J, Takemura M, Misawa H, Ikawa M (2004). The LIM homeobox gene, L3/Lhx8, is necessary for proper development of basal forebrain cholinergic neurons. Eur J Neurosci.

[23] Asbreuk CH, van Schaick HS, Cox JJ, Kromkamp M, Smidt MP, Burbach JP (2002). The homeobox genes Lhx7 and Gbx1 are expressed in the basal forebrain cholinergic system. Neuroscience.

[24] Manabe T, Tatsumi K, Inoue M, Makinodan M, Yamauchi T, Makinodan E, Yokoyama S, Sakumura R, Wanaka A (2007). L3/Lhx8 is a pivotal factor for cholinergic differentiation of murine embryonic stem cells. Cell Death Differ.

[25] Zhao Y, Marin O, Hermesz E, Powell A, Flames N, Palkovits M, Rubenstein JL, Westphal H (2003). The LIM-homeobox gene Lhx8 is required for the development of many cholinergic neurons in the mouse forebrain. Proc Natl Acad Sci U S A.

[26] Fragkouli A, Hearn C, Errington M, Cooke S, Grigoriou M, Bliss T, Stylianopoulou F, Pachnis V (2005). Loss of forebrain cholinergic neurons and impairment in spatial learning and memory in LHX7-deficient mice. Eur J Neurosci.

[27] Ma YC, Song MR, Park JP, Henry Ho HY, Hu L, Kurtev MV, Zieg J, Ma Q, Pfaff SL, Greenberg ME (2008). Regulation of motor neuron specification by phosphorylation of neurogenin 2. Neuron.

[28] Mizuguchi R, Sugimori M, Takebayashi H, Kosako H, Nagao M, Yoshida S, Nabeshima Y, Shimamura K, Nakafuku M (2001). Combinatorial roles of olig2 and neurogenin2 in the coordinated induction of pan-neuronal and subtype-specific properties of motoneurons. Neuron.

[29] Herdy J, Schafer S, Kim Y, Ansari Z, Zangwill D, Ku M, Paquola A, Lee H, Mertens J, Gage FH (2019). Chemical modulation of transcriptionally enriched signaling pathways to optimize the conversion of fibroblasts into neurons. Elife.

[30] Ladewig J, Mertens J, Kesavan J, Doerr J, Poppe D, Glaue F, Herms S, Wernet P, Kogler G, Muller FJ (2012). Small molecules enable highly efficient neuronal conversion of human fibroblasts. Nat Methods.

[31] Kim EJ, Battiste J, Nakagawa Y, Johnson JE (2008). Ascl1 (Mash1) lineage cells contribute to discrete cell populations in CNS architecture. Mol Cell Neurosci.

[32] Marin O, Anderson SA, Rubenstein JL (2000). Origin and molecular specification of striatal interneurons. J Neurosci.

[33] Liang XG, Tan C, Wang CK, Tao RR, Huang YJ, Ma KF, Fukunaga K, Huang MZ, Han F (2018). Myt1l induced direct reprogramming of pericytes into cholinergic neurons. CNS Neurosci Ther.

[34] Vetreno RP, Crews FT (2018). Adolescent binge ethanol-induced loss of basal forebrain cholinergic neurons and neuroimmune activation are prevented by exercise and indomethacin. PLoS One.

[35] Insua D, Corredoira A, Gonzalez-Martinez A, Suarez ML, Santamarina G, Sarasa M, Pesini P (2012). Expression of p75(NTR), a marker for basal forebrain cholinergic neurons, in young and aged dogs with or without cognitive dysfunction syndrome. J Alzheimers Dis.

[36] Yeo TT, Chua-Couzens J, Butcher LL, Bredesen DE, Cooper JD, Valletta JS, Mobley WC, Longo FM (1997). Absence of p75NTR causes increased basal forebrain cholinergic neuron size, choline acetyltransferase activity, and target innervation. J Neurosci.

[37] Mufson EJ, Ginsberg SD, Ikonomovic MD, DeKosky ST (2003). Human cholinergic basal forebrain: chemoanatomy and neurologic dysfunction. J Chem Neuroanat.

[38] Woolf NJ, Gould E, Butcher LL (1989). Nerve growth factor receptor is associated with cholinergic neurons of the basal forebrain but not the pontomesencephalon. Neuroscience.

[39] Pioro EP, Cuello AC (1990). Distribution of nerve growth factor receptor-like immunoreactivity in the adult rat central nervous system. Effect of colchicine and correlation with the cholinergic system--I. forebrain. Neuroscience.

[40] Allaway KC, Machold R (2017). Developmental specification of forebrain cholinergic neurons. Dev Biol.

[41] Elshatory Y, Gan L (2008). The LIM-homeobox gene Islet-1 is required for the development of restricted forebrain cholinergic neurons. J Neurosci.

[42] Cho HH, Cargnin F, Kim Y, Lee B, Kwon RJ, Nam H, Shen R, Barnes AP, Lee JW, Lee S, Lee SK (2014). Isl1 directly controls a cholinergic neuronal identity in the developing forebrain and spinal cord by forming cell type-specific complexes. PLoS Genet.

[43] Minc E, Courvalin JC, Buendia B (2000). HP1gamma associates with euchromatin and heterochromatin in mammalian nuclei and chromosomes. Cytogenet Cell Genet.

[44] Capsoni S, Tiveron C, Vignone D, Amato G, Cattaneo A (2010). Dissecting the involvement of tropomyosin-related kinase a and p75 neurotrophin receptor signaling in NGF deficit-induced neurodegeneration. Proc Natl Acad Sci U S A.

[45] Capsoni S, Amato G, Vignone D, Criscuolo C, Nykjaer A, Cattaneo A (2013). Dissecting the role of sortilin receptor signaling in neurodegeneration induced by NGF deprivation. Biochem Biophys Res Commun.

[46] Moreau K, Fleming A, Imarisio S, Lopez Ramirez A, Mercer JL, Jimenez-Sanchez M, Bento CF, Puri C, Zavodszky E, Siddiqi F (2014). PICALM modulates autophagy activity and tau accumulation. Nat Commun.

[47] Braak H, Del Tredici K (2011). The pathological process underlying Alzheimer's disease in individuals under thirty. Acta Neuropathol.

[48] Trzeciakiewicz H, Tseng JH, Wander CM, Madden V, Tripathy A, Yuan CX, Cohen TJ (2017). A dual pathogenic mechanism links tau acetylation to sporadic Tauopathy. Sci Rep.

[49] Ohia-Nwoko O, Montazari S, Lau YS, Eriksen JL (2014). Long-term treadmill exercise attenuates tau pathology in P301S tau transgenic mice. Mol Neurodegener.

[50] Choi SH, Kim YH, Hebisch M, Sliwinski C, Lee S, D'Avanzo C, Chen H, Hooli B, Asselin C, Muffat J (2014). A three-dimensional human neural cell culture model of Alzheimer's disease. Nature.

[51] Eftekharzadeh B, Daigle JG, Kapinos LE, Coyne A, Schiantarelli J, Carlomagno Y, Cook C, Miller SJ, Dujardin S, Amaral AS (2018). Tau protein disrupts Nucleocytoplasmic transport in Alzheimer's disease. Neuron.

[52] Ding B, Akter M, Zhang CL (2020). Differential influence of sample sex and neuronal maturation on mRNA and protein transport in induced human neurons. Front Mol Neurosci.

[53] Bartus RT, Dean RL, Beer B, Lippa AS (1982). The cholinergic hypothesis of geriatric memory dysfunction. Science.

[54] Perry EK, Tomlinson BE, Blessed G, Bergmann K, Gibson PH, Perry RH (1978). Correlation of cholinergic abnormalities with senile plaques and mental test scores in senile dementia. Br Med J.

[55] Smith DK, Yang J, Liu ML, Zhang CL (2016). Small molecules modulate chromatin accessibility to promote NEUROG2-mediated fibroblast-to-neuron reprogramming. Stem Cell Reports.

[56] Wapinski OL, Vierbuchen T, Qu K, Lee QY, Chanda S, Fuentes DR, Giresi PG, Ng YH, Marro S, Neff NF (2013). Hierarchical mechanisms for direct reprogramming of fibroblasts to neurons. Cell.

[57] Thaler JP, Lee SK, Jurata LW, Gill GN, Pfaff SL (2002). LIM factor Lhx3 contributes to the specification of motor neuron and interneuron identity through cell-type-specific protein-protein interactions. Cell.

[58] Galluzzi L, Kepp O, Krautwald S, Kroemer G, Linkermann A (2014). Molecular mechanisms of regulated necrosis. Semin Cell Dev Biol.

[59] Fricker M, Tolkovsky AM, Borutaite V, Coleman M, Brown GC (2018). Neuronal cell death. Physiol Rev.

