# Supplementary material for: Aging-relevant human basal forebrain cholinergic neurons as a cell model for Alzheimer’s disease
Source: Mol Neurodegener. 2020 Oct 21;15:61. doi: 10.1186/s13024-020-00411-6 (PMC7579825; doi:10.1186/s13024-020-00411-6)
Supplement: Supplementary file 1 — Additional file 1: Figure S1. The reprogramming process and optimization, related to Fig. 1 A. [file 13024_2020_411_MOESM1_ESM.pdf]

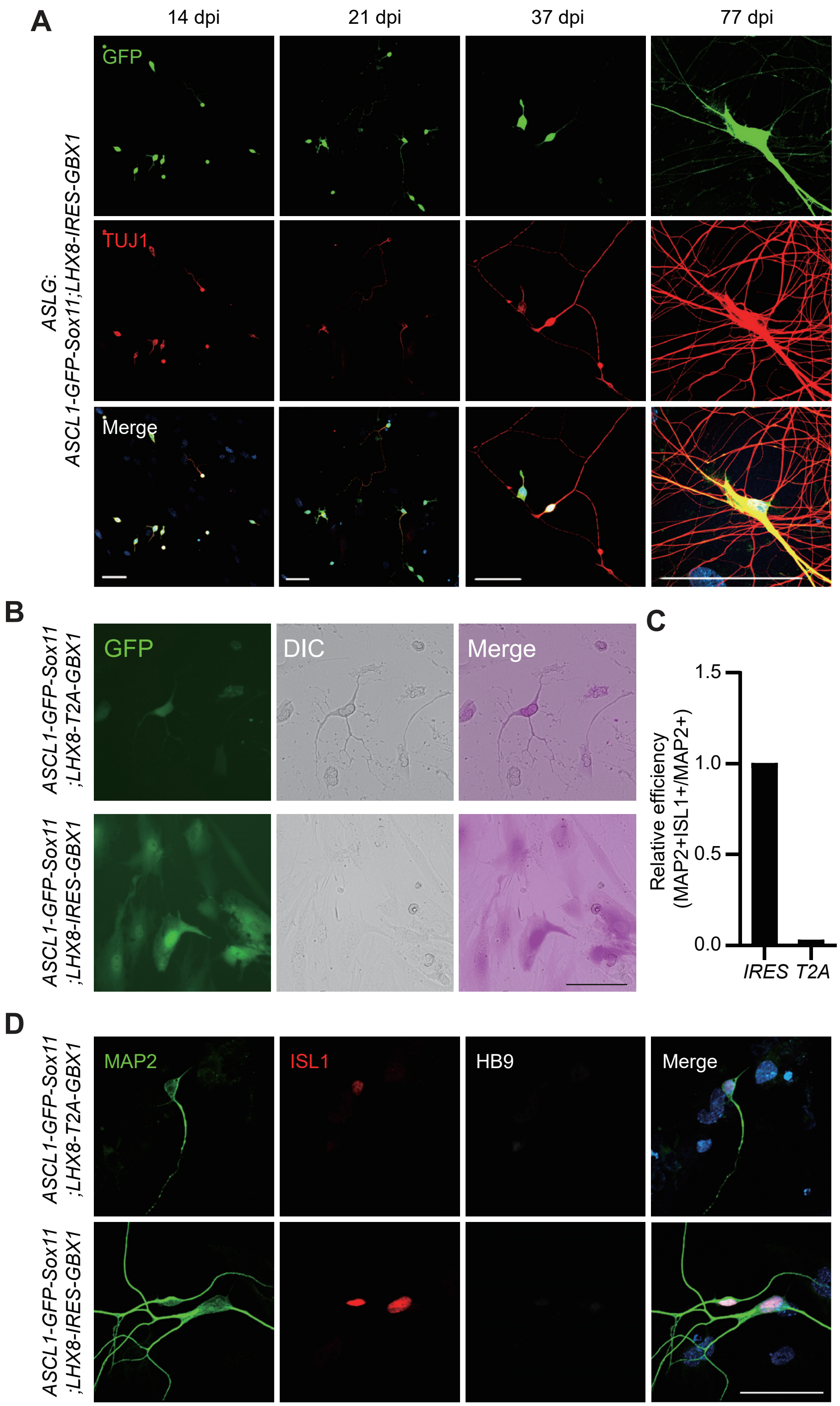

Figure S1. The reprogramming process and optimization, related to Fig. 1

A. Confocal images of induced neurons at the indicated time points. Scale bars: 100 μm.

B. Representative images of fibroblasts transduced with virus expressing the indicated reprogramming factors at 4 dpi. Cell morphology is also shown under DIC illumination. Scale bar, 100 μm.

C. Quantification of induced neurons with expression of the indicated markers at 28 dpi. *IRES*, *LHX8-IRES-GBX1* and *ASCL1-GFP-Sox11*; *T2A*, *LHX8-T2A-GBX1* and *ASCL1-GFP-Sox11*.

D. Confocal images of induced neurons by the indicated transcription factors at 28 dpi. Scale bar, 100 μm.
