# Supplementary material for: Aging-relevant human basal forebrain cholinergic neurons as a cell model for Alzheimer’s disease
Source: Mol Neurodegener. 2020 Oct 21;15:61. doi: 10.1186/s13024-020-00411-6 (PMC7579825; doi:10.1186/s13024-020-00411-6)
Supplement: Supplementary file 2 — Additional file 2: Figure S2. Characterization of the induced neurons, related to Fig. 1 A. [file 13024_2020_411_MOESM2_ESM.pdf]

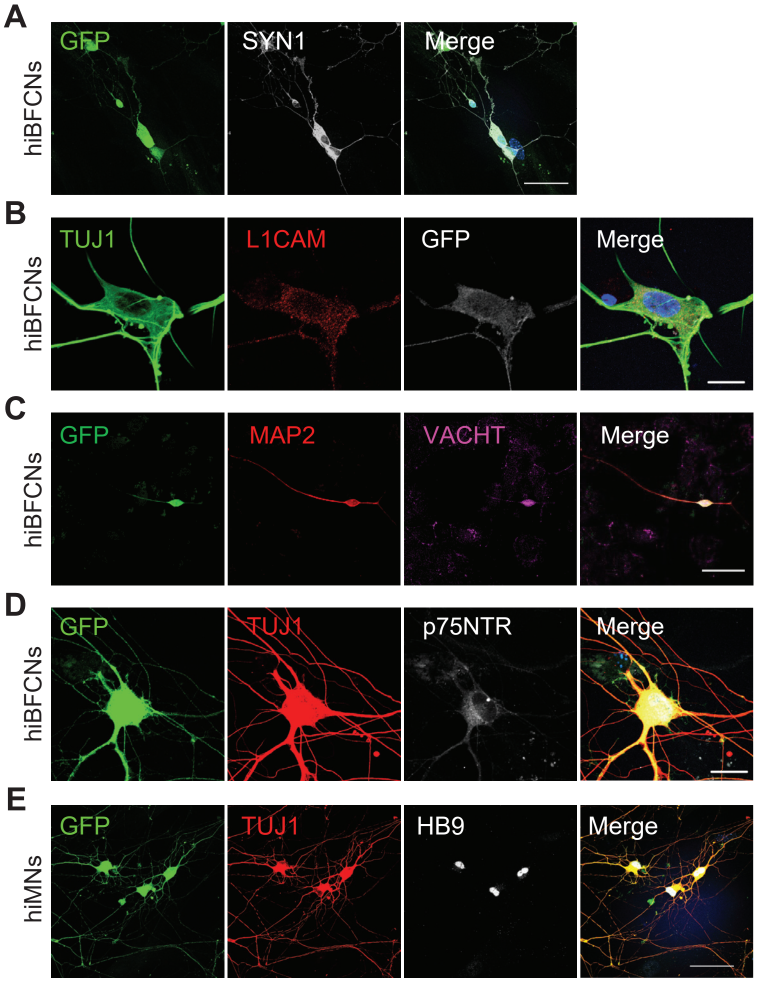

Figure S2. Characterization of the induced neurons, related to Fig. 1

A. Confocal images of the indicated marker SYN1 in hiBFCNs at 28 dpi. Scale bar, 50 µm.
B. Confocal images of the indicated marker L1CAM in hiBFCNs at 75 dpi. Scale bar, 20 µm.
C. Confocal images of the indicated marker VACHT in hiBFCNs at 28 dpi. Scale bar, 50 µm.
D. Confocal images of the indicated marker p75NTR in hiBFCNs at 77 dpi. Scale bar, 20 µm.
E. HB9 expression in human induced motor neurons (hiMNs) at 28 dpi. Scale bar, 50 µm.
