# Supplementary material for: Aging-relevant human basal forebrain cholinergic neurons as a cell model for Alzheimer’s disease
Source: Mol Neurodegener. 2020 Oct 21;15:61. doi: 10.1186/s13024-020-00411-6 (PMC7579825; doi:10.1186/s13024-020-00411-6)
Supplement: Supplementary file 3 — Additional file 3: Figure S3. Characterization of AD-hiBFCNs, related to Fig. 1 A. [file 13024_2020_411_MOESM3_ESM.pdf]

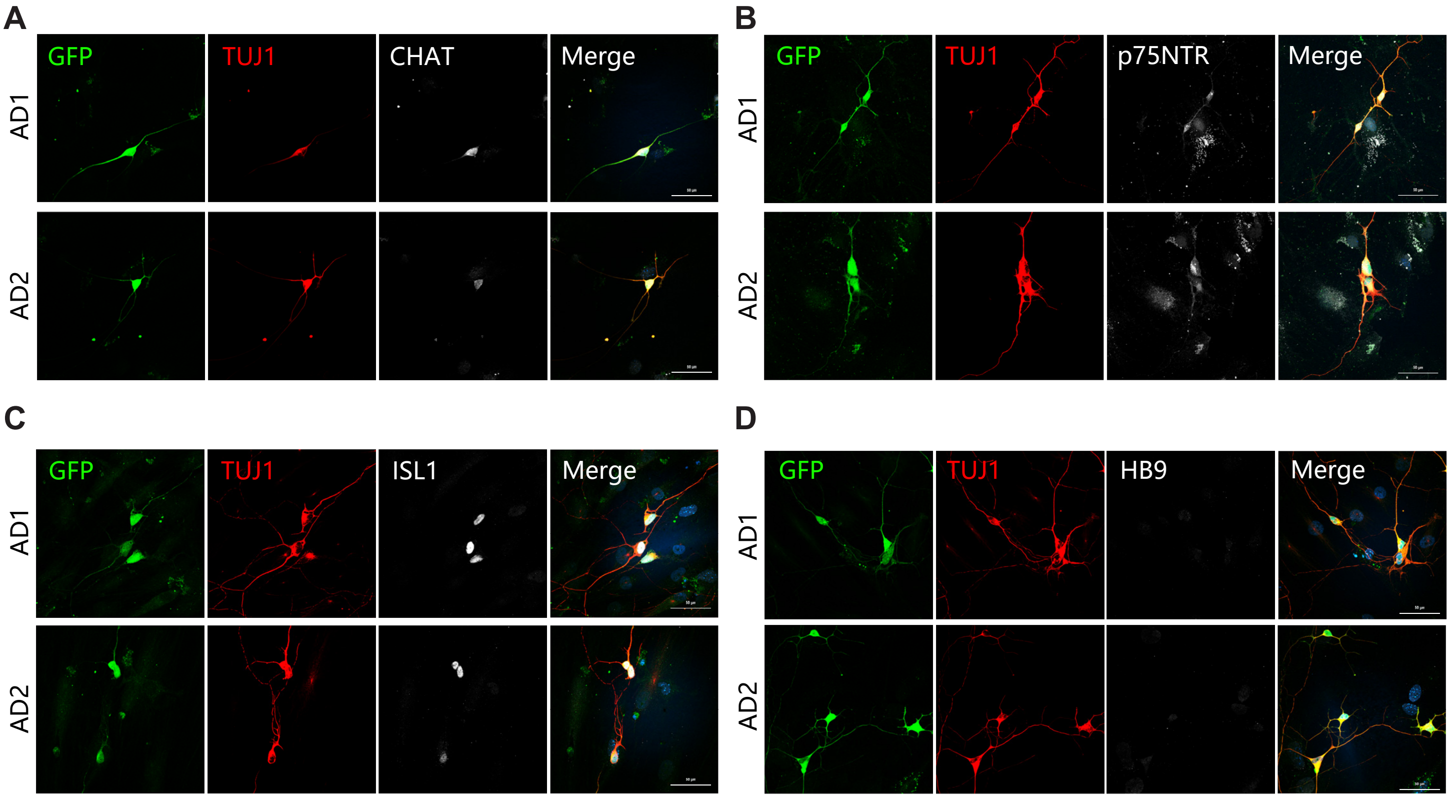

Figure S3. Characterization of AD-hiBFCNs, related to Fig. 1

A. Confocal images of the indicated marker CHAT in AD-hiBFCNs at 28 dpi. Scale bar, 50 µm.

B. Confocal images of the indicated marker p75NTR in AD-hiBFCNs at 28 dpi. Scale bar, 50 µm.

C. Confocal images of the indicated marker ISL1 in AD-hiBFCNs at 28 dpi. Scale bar, 50 µm.

D. hiBFCNs do not express HB9, a marker restricted to cholinergic motor neurons. Scale bar, 50 µm.
