# Supplementary material for: Aging-relevant human basal forebrain cholinergic neurons as a cell model for Alzheimer’s disease
Source: Mol Neurodegener. 2020 Oct 21;15:61. doi: 10.1186/s13024-020-00411-6 (PMC7579825; doi:10.1186/s13024-020-00411-6)
Supplement: Supplementary file 5 — Additional file 5: Figure S5. Effect of LMB on nucleocytoplasmic transport in hiBFCNs, related to Fig. 6 A. [file 13024_2020_411_MOESM5_ESM.pdf]

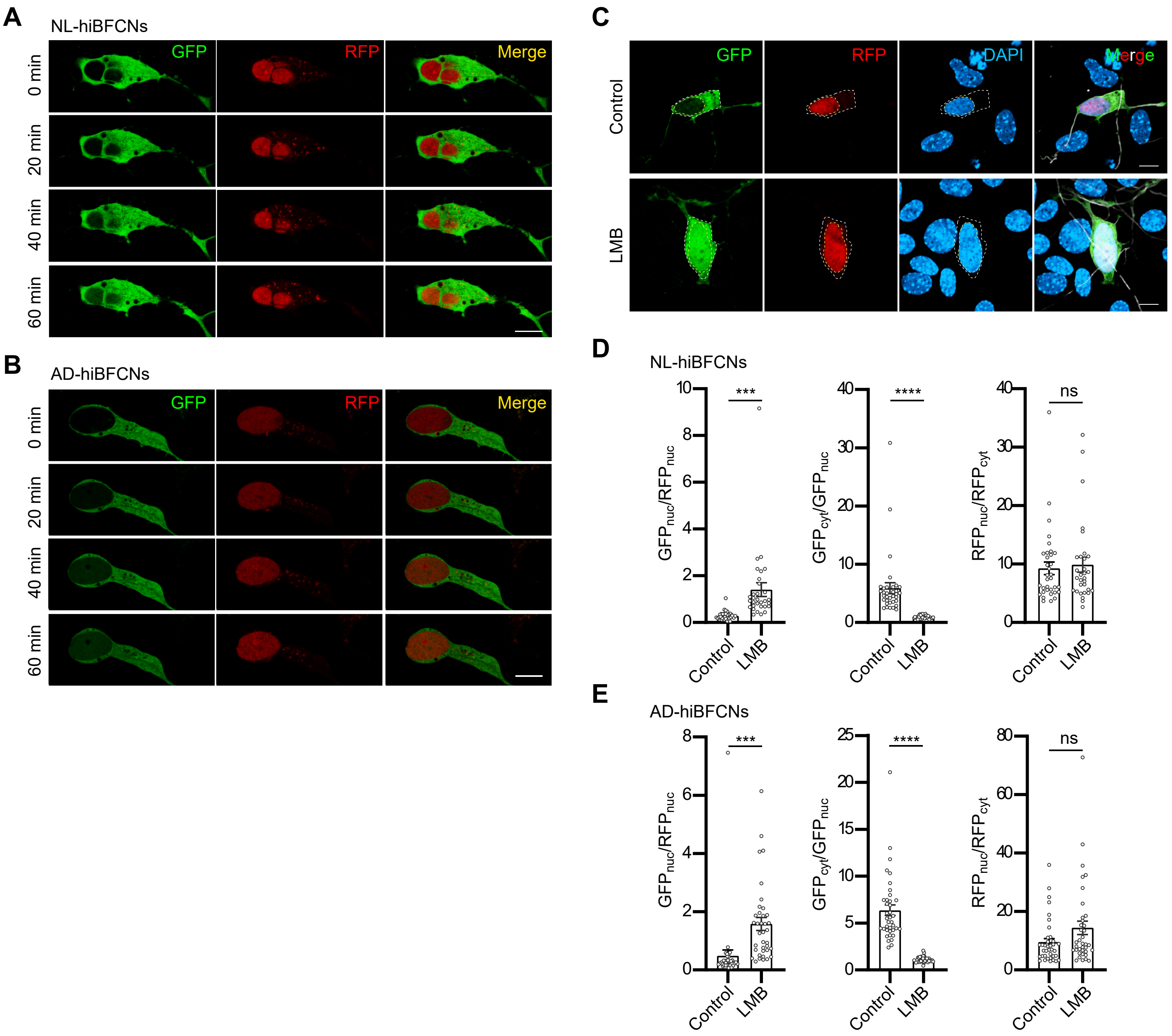

Figure S5. Effect of LMB on nucleocytoplasmic transport in hiBFCNs, related to Fig. 6

A, B. Representative live-cell confocal images of LMB-treated hiBFCNs at the indicated time points. hiBFCNs were co-cultured with astrocytes until 51 dpi and treated with 50 nM LMB. The 2Gi2R reporter was imaged every 10 min for 1 h. Scale bars: 10 μm.

C. Representative confocal images of reporter subcellular distributions in hiBFCNs with or without LMB treatment at 51 dpi.

D. Quantitative analysis of the reporter subcellular distributions in NL-hiBFCNs at 51 dpi. Each point represents a single cell (mean ± SEM; n = 34 for control group; n = 32 for LMB group; ***p = 0.0001, and ****p < 0.0001).

E. Quantification of subcellular distributions of the reporters in AD-hiBFCNs at 51 dpi. Each point represents a single cell (mean ± SEM; n = 38 for control group; n = 38 for LMB group; ***p = 0.0004, and ****p < 0.0001).
