# Supplementary material for: Aging-relevant human basal forebrain cholinergic neurons as a cell model for Alzheimer’s disease
Source: Mol Neurodegener. 2020 Oct 21;15:61. doi: 10.1186/s13024-020-00411-6 (PMC7579825; doi:10.1186/s13024-020-00411-6)
Supplement: Supplementary file 6 — Additional file 6: Table S1. Primary antibodies. Table S2. Primer sequences. [file 13024_2020_411_MOESM6_ESM.pdf]

Table S1. Primary antibodies.

Antibodies	Application and Dilution	Source	Cat#
Chicken anti-GFP	IF 1:1,000	AVES	879484
Rabbit anti-RFP	IF 1:1,000	Clontech	632496
Rabbit anti-TUJ1	IF 1:4,000	Covance	802001
Mouse anti-TUJ1	IF 1:4,000	Covance	801202
Chicken anti-MAP2	IF 1:10,000	Abcam	ab5392
Rabbit anti-NF200	IF 1:1,000	Sigma	N4142
Rabbit anti-SYN1	IF 1:2,000	Cell Signaling	#5297
Mouse anti-HB9	IF 1:500	DSHB	81.5C10
Goat anti-CHAT	IF 1:100	Chemicon	AP144P
Rabbit anti-VACHT	IF 1:2,000	Synaptic Systems	139103
Mouse anti-γH2AX	IF 1:500	Millipore	#05-636
Rabbit anti-H3K9me3	IF 1:5,000	Abcam	ab8898
Rabbit anti- HP1γ	IF 1:200	Cell Signaling	#2619
Mouse anti-L1CAM	IF 1:20	Millipore	CBL275
Rabbit anti-p75NTR	IF 1:500	Abcam	ab8874
Rabbit anti-ISL1	IF 1:200	Abcam	Ab109517
Mouse anti-AT8	IF 1:500; WB 1:10,000 (in 5% BSA)	Invitrogen	MN1020
Chicken anti-TAU	WB 1:20,000 (in 5% BSA)	AVES	TAU

Table S2. Primer sequences

Gene Name	Sequence (5'-3')	
	**Forward primer**	**Reverse primer**
GAPDH	*GTCAAGGCTGAGAACGGGAA*	*AAATGAGCCCCAGCCTTCTC*
MAPT	*CCAAGTGTGGCTCATTAGGCA*	*CCAATCTTCGACTGGACTCTGT*
VACHT	*TTCGCCTCTACAGTCCTGTTC*	*GCTCCTCCGGGTACTTATCG*
NKX2.1	*AGCACACGACTCCGTTCTC*	*GCCCACTTTCTTGTAGCTTTCC*
TRKA	*AACCTCACCATCGTGAAGAGT*	*TGAAGGAGAGATTCAGGCGAC*
ACHE	*GGGTGGTAGACGCTACAACC*	*GTGCCCTCAAAACCTGGGTAT*
CALB1	*GGCTCCATTTCGACGCTGA*	*GCCCATACTGATCCACAAAAGTT*
MAP2	*GCTCTGCCTTTAGCAGCTGAA*	*GTCTGTTCTGAGGCAGGTGATG*
CHAT	*GCACTCCAGCTCCTTCAC*	*CACTGCACCAGGACGATG*
ISL1	*TCAATGTCCTCTCAACTTCCAG*	*TTCCCACTTTCTCCAACAGG*
HB9	*GCACCAGTTCAAGCTCAAC*	*GCTGCGTTTCCATTTCATCC*
S100A4	*GATGAGCAACTTGGACAGCAA*	*CTGGGCTGCTTATCTGGGAAG*
VIM	*AGTCCACTGAGTACCGGAGAC*	*CATTTCACGCATCTGGCGTTC*
