# Supplementary material for: Aging-relevant human basal forebrain cholinergic neurons as a cell model for Alzheimer’s disease
Source: Mol Neurodegener. 2020 Oct 21;15:61. doi: 10.1186/s13024-020-00411-6 (PMC7579825; doi:10.1186/s13024-020-00411-6)
Supplement: Supplementary file 4 — Additional file 4: Figure S4. PhosphoTAU expression in hiBFCNs, related to Fig. 5 A. [file 13024_2020_411_MOESM4_ESM.pdf]

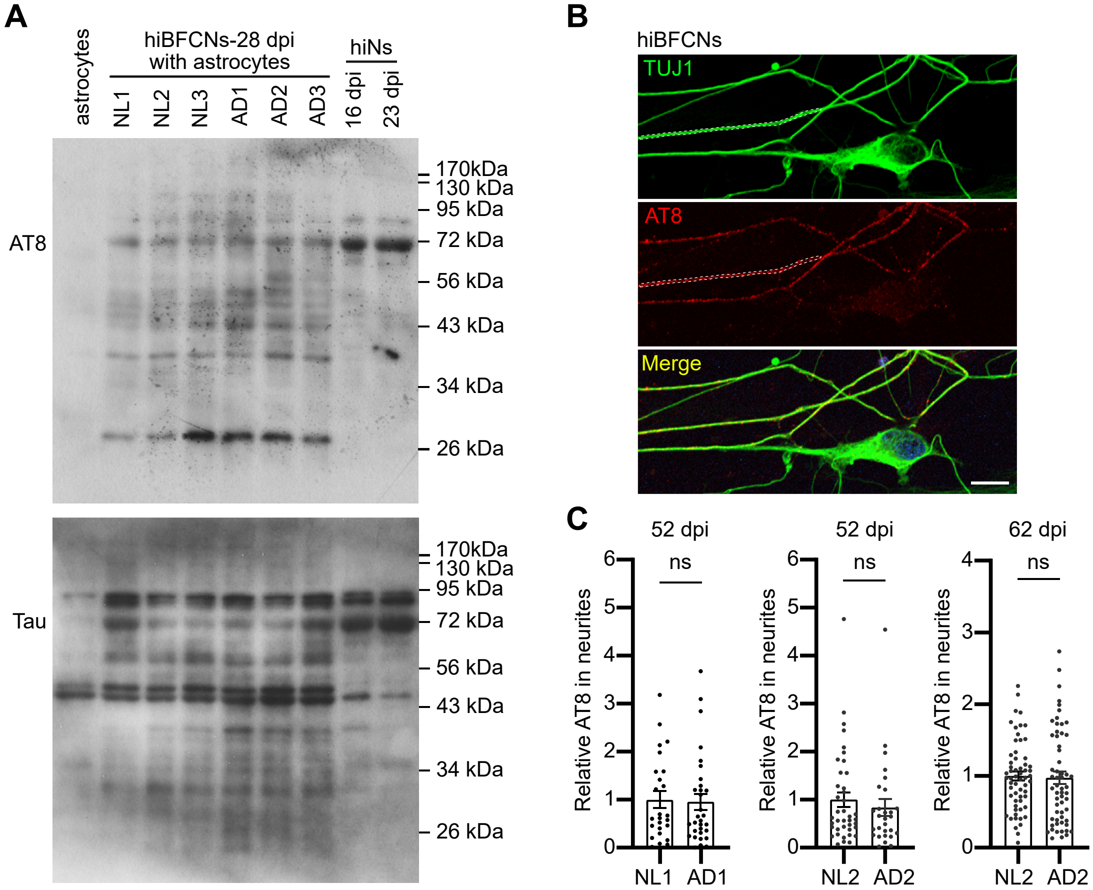

Figure S4. PhosphoTAU expression in hiBFCNs, related to Fig. 5

A. Western blots of phosphoTAU (AT8) and total TAU in hiBFCNs. hiBFCNs were co-cultured with astrocytes, whereas the control hiNs from healthy fibroblasts were cultured alone. Astrocytes were also included as a negative control. Cells at the indicated time-points were collected for analysis.

B. Representative confocal images for AT8 expression in neurites of hiBFCNs. A representative area used for quantification is outlined. Scale bar, 10 µm.

C. No significant difference of neuritic AT8 expression among the indicated hiBFCNs. Each point represents a single measurement from 50-µm length of neurite.
